# PCSK9 promotes atherosclerosis progression through the FOXO3a autophagy signaling pathway

**DOI:** 10.3389/fcvm.2026.1836294

**Published:** 2026-06-15

**Authors:** Yuanjia Shi, Jing Zhang, Lina Dai, Jie Chen, Shijia Geng, Yan Niu, Chongyang Dong, Chenlei Li, Rujin Liu, Ningxia Zhao, Xiaomeng Wang, Zhanfeng Gao, Xi Yang, Shang Gao

**Affiliations:** 1 Department of Basic Medicine, School of Basic Medical Sciences, Inner Mongolia Medical University, Hohhot, China; 2 College of Basic Medicine, Inner Mongolia Medical University, Hohhot, China; 3 Medical Experiments Center, Inner Mongolia Medical University, Hohhot, China; 4 College of Traditional Chinese Medicine, Inner Mongolia Medical University, Hohhot, China; 5 Department of Cardiology, The Affiliated Hospital, Inner Mongolia Medical University, Hohhot, China

**Keywords:** atherosclerosis, autophagy, COS, FOXO3a, PCSK9

## Abstract

**Objectives:**

The proprotein convertase subtilisin/kexin type 9 (PCSK9) has an impact on atherosclerosis by modulating the autophagy pathway; however, its precise mechanisms underlying autophagy require additional investigation.

**Methodology and results:**

ApoE−/− male mice were fed high-fat diets, different doses of COS (Chitosan oligosaccharide) or JY2 were given by gavage, and RAPA or CQ was injected intraperitoneally. The immunofluorescence intensities of PCSK9, FOXO3a and LC3, collagen fibers and calcium salt deposition in aortic sinus tissues were measured. *In vitro*, macrophages were pretreated with oe/siPCSK9, COS, RAPA, CQ or JY2 combined with ox-LDL, and autophagy-related protein levels, autophagic flux and autophagy-related genes were detected. COS significantly reduced PCSK9 expression in Raw264.7 cells and autophagy-related protein expression, slowing the progression of atherosclerotic plaques, as shown by reduced plaque area, lipid deposition, and calcium salt deposition and increased collagen fibers. In macrophages derived from atherosclerotic plaques, the concentrations of PCSK9 and FOXO3a were markedly elevated in the RAPA group compared to the ND group. *In vitro* studies revealed that PCSK9 enhances the expression of Beclin1, P62, LC3, and FOXO3a, while also augmenting autophagic flux, thereby promoting ox-LDL-induced autophagic injury in macrophages, whereas COS inhibited the formation of PCSK9, thereby inhibiting autophagic injury. In addition, the FOXO3a inhibitor JY2 significantly inhibited autophagy but did not significantly affect PCSK9. In addition, oe/siPCSK9 affected the extent and flow of autophagy in macrophages. COS and CQ also limit PCSK9 in ApoE−/− mouse atherosclerosis progression and in macrophages subjected to autophagy.

**Conclusions and significance:**

PCSK9 promotes macrophage autophagy injury and facilitates atherosclerosis progression through activation of the FOXO3a autophagy pathway, whereas COS reduces PCSK9 injury to macrophages and atherosclerosis. These findings provide a theoretical basis and experimental foundation for the future development of PCSK9 inhibitors as a new strategy for the treatment of As.

## 1 Introduction

Atherosclerosis (As) is a progressive arterial disease (1). A key process in its development involves the uptake of lipoproteins and cellular debris by macrophages beneath the endothelium, leading to the formation of foam cells and the initiation of an inflammatory response (2). Proprotein convertase subtilisin/kexin type 9 (PCSK9) inhibitors represent a novel class of lipid-lowering drugs that effectively reduce low-density lipoprotein (LDL) cholesterol levels and mitigate atherosclerosis risk. Recent studies indicate PCSK9 expression correlates with autophagy development, suggesting PCSK9 may regulate this cellular process (3–6). Notably, treating Raw264.7 cells with PCSK9 siRNA significantly attenuated the autophagic process, underscoring PCSK9's influence on this pathway. Furthermore, PCSK9 expression and release correlate with the forkhead box O3a (FOXO3a)-mediated autophagy signaling pathway (7). Studies highlight FOXO3a's role in activating autophagy by directly binding to the promoters of autophagy-related genes, thereby influencing the progression of atherosclerosis.

Autophagy is a membrane-mediated biological process that engulfs cytoplasmic components for subsequent degradation within lysosomes. Recent evidence indicates autophagy plays a critical role in both early and late stages of atherosclerotic plaque formation (8, 9). The autophagy process is regulated by multiple signaling pathways, including FOXO3a-mediated induction pathways and the synergistic action of autophagy-related genes (ATGs) during autophagosome formation. In both human and mouse vascular tissues, impaired chaperone-mediated autophagy has been observed during atherosclerotic progression. This deficiency exacerbates excessive inflammasome activation, intensifies vascular inflammation, and promotes atherosclerosis (10, 11). Therefore, activating the autophagy-lysosome pathway holds promise as a therapeutic strategy to control this condition (12). Furthermore, excessive autophagy activation can also trigger programmed cell death, potentially influencing atherosclerosis development (13). Autophagy-related proteins (ATGs) form unique complexes responsible for altering membrane dynamics and facilitating cargo uptake and degradation (14). Under normal conditions, the core autophagy protein LC3 exists as LC3-I. Following autophagy activation, LC3-I converts to LC3-II, which is associated with autophagosome formation (15). Furthermore, both LC3-II and P62 serve as key autophagy adaptor proteins involved in protein degradation and are routinely assessed to monitor cellular autophagy dynamics (16). This analysis aids in evaluating autophagy flux efficiency and provides insights into how these compounds influence the autophagy pathway (17). Beclin1 is another crucial regulator playing a key role in vesicle nucleation during autophagosome formation (18). Collectively, these findings underscore that autophagy has emerged as a critical mechanism influencing late-stage atherosclerosis.

PCSK9 expression can be regulated through LDL receptor-mediated hepatic uptake of apoB lipoprotein and serine protease-mediated cholesterol metabolism (19–21). Consequently, PCSK9-targeting inhibitors have emerged as therapeutic agents for controlling lipid-related diseases such as diabetes, acute coronary syndrome, stroke, and hypercholesterolemia (22). Recent studies indicate that PCSK9 may significantly inhibit cholesterol degradation and promote Raw264.7 foam cell formation by engaging the autophagy signaling pathway involving PI3K-AKT-mTOR-FOXO3a. FOXO3a plays a pivotal role in regulating numerous cellular processes and is crucial for maintaining cellular homeostasis and influencing disease progression (23–25). FOXO3a is closely associated with autophagy (26), participating in the transcriptional regulation of autophagy-related genes including Beclin1, ATG12, ULK1, P62, ATG5, ATG7, and LC3B (27). Activation of FOXO3a induces autophagy in Raw264.7 cells, contributing to enhanced lipid accumulation, an effect partially attributable to PCSK9 activity. Relevant studies indicate that PCSK9 significantly elevates levels of the FOXO3a protein, enhances the formation of the FOXO3a/PCSK9 promoter complex, and diminishes the nuclear HNF-1α promoter binding capacity (7, 16, 28). This finding provides theoretical support for PCSK9 inhibitors influencing atherosclerosis by regulating FOXO3a levels. Recent studies further reveal that PCSK9 may significantly inhibit cholesterol degradation and promote the transformation of Raw264.7 macrophages into foam cells via the PI3K-AKT-mTOR-FOXO3a autophagy signalling pathway (29–33). These findings not only confirm FOXO3a's role in activating autophagy by directly binding to autophagy-related gene promoters, but also provide novel insights into the molecular mechanisms by which PCSK9 regulates the progression of atherosclerosis. FOXO3a activation promotes atherosclerosis and autophagy, potentially exacerbating vascular endothelial injury in a PCSK9-dependent manner. Interestingly, chitin oligosaccharides (COS) can in turn influence PCSK9 levels. These findings stimulate further investigation into the interaction between the autophagy signaling pathways controlled by FOXO3 and PCSK9, and how their interplay affects atherosclerosis progression.

In recent years, chitin oligosaccharides (COS) have garnered attention as a potential therapeutic agent. Research indicates that COS effectively alleviates oxidative liver damage through its core mechanism of activating the Nrf2 signaling pathway. Furthermore, COS demonstrates promising applications in atherosclerosis via its antioxidant, anti-inflammatory, and multifaceted mechanisms (34). COS elevates serum high-density lipoprotein cholesterol (HDL-C) levels, improving cardiovascular risk factors by promoting cholesterol reverse transport (35–38). This study found that in an ox-LDL-induced Raw264.7 foam cell model, COS post-treatment not only significantly downregulated PCSK9 expression but also reduced the expression levels of autophagy-related proteins (such as LC3-II and Beclin1) by inhibiting the FOXO3a signaling pathway. These results confirm that COS effectively inhibits abnormal autophagosome accumulation in foam cells by regulating the PCSK9-FOXO3a-autophagy signaling axis, thereby delaying the pathological progression of atherosclerosis. However, this study has limitations, as the interaction mechanism between PCSK9 and FOXO3a remains incompletely explored and requires further clarification in future research. Furthermore, exploring the effects of modulating PCSK9 or FOXO3a levels during As development on oxidative stress, cell migration capacity, apoptosis, inflammatory mechanisms, or therapeutic strategies represents an important future research direction. Current data indicate that PCSK9 promotes lipid accumulation, calcium salt deposition, reduced collagen fibers, and macrophage formation in As through autophagy activation, while COS inhibits PCSK9-induced increased autophagy and atherosclerotic plaque formation.

In summary, these findings indicate that PCSK9 enhances autophagy in ox-LDL-induced Raw264.7 macrophages by regulating the FOXO3a/autophagy signaling pathway. In this study, the functional role of autophagy in Raw264.7 macrophages was examined by altering PCSK9 expression levels. The upstream-downstream relationship between PCSK9 and FOXO3a was assessed using a FOXO3a inhibitor (JY2), and the impact of the PCSK9/FOXO3a signaling pathway on atherosclerosis (As) development was validated using COS. By controlling PCSK9 levels, the study elucidated its effects on the FOXO3a autophagy mechanism and As, providing insights into potential mechanisms underlying As progression.

## 2 Materials and methods

### 2.1 Reagents and antibodies

Chloroquine (CQ, MedChemExpress, HY-17589A), rapamycin (RAPA, MedChemExpress, HY-10219), JY2 (JY-2, MedChemExpress, HY - 153347), and ox-LDL (yi yuan biotechnology company, Guangzhou, China) were used. The following related antibodies were used: PCSK9(Affbiotech, Jiangsu, China) P62 (Santa Cruz Biotechnology, sc-28359), Beclin1 (Santa Cruz Biotechnology, Sc-48341), p-FOXO3a (Proteintech, Wuhan, China, 28755-1-AP) and TLR4 (Santa Cruz Biotechnology, sc-293072). For F4/80 (Abcam, ab6640, UK), Western blot analysis revealed two types of species-specific resistance: mouse (Proteintech, Wuhan, China) and rabbit (Proteintech, Wuhan, China). In secondary antibody immunofluorescence staining, the following reagents were utilized: Cy3-labeled goat anti-rabbit IgG (Servicebio, GB21303), Alexa Fluor 488-conjugated anti-mouse IgG (Servicebio, GB25301), and Fluor 350-conjugated anti-rat IgG (Bioss, bs-0346R-BF350). A Masson staining kit (Solarbio, G-1346) and an Alizarin red staining kit (Solarbio, G-1452) were used.

### 2.2 Animals

Male ApoE−/− mice (8 weeks old) were obtained from the Laboratory Animal Center of Inner Mongolia Medical University. The mice were then randomly divided into eight groups, each containing eight ApoE−/− mice, and an atherosclerosis mouse model was established using a high-fat diet (0.15% cholesterol; 21% fat; HFK Biological Sciences, Beijing, China). They were divided into ND group (normal diet, 12 weeks normal diet, *n* = 8). To dynamically observe the relationship between advanced atherosclerosis and autophagy, this study constructed atherosclerosis animal models with varying durations (14–19 weeks), high-fat diet feeding was uninterrupted during this period. 14W group (baseline group, 14 weeks high-fat diet, *n* = 8); 19W group (progression group, 19 weeks high-fat diet, *n* = 8). The other five groups continued to eat high-fat diet until the 14th week as the experimental group and then were administered for 4 weeks and randomly assigned to the CQ group (autophagy inhibitor, intraperitoneal injection, 50 mg/kg/2 days, dissolved in DMSO, *n* = 8), the RAPA group (autophagy agonist, intraperitoneal injection, 20 mg/kg/2 days, dissolved in DMSO, *n* = 8), JY2 group (FOXO3a inhibitor, orally administered, 50 mg/kg/2 days, dissolved in DMSO, *n* = 8), Given the extremely low DMSO concentration in this formulation (10%), which falls within the safety threshold (≤10%), and considering that it is rapidly diluted *in vivo* following intraperitoneal injection or oral administration, its physiological effects are negligible. Therefore, a separate DMSO solvent control group was not established. HCOS group (high COS, orally administered, 500 mg/kg/days, dissolved in PBS, *n* = 8) and LCOS group (low COS, orally administered, 200 mg/kg/days, dissolved in PBS, *n* = 8).

All animals were housed at the Experimental Animal Center of Inner Mongolia Medical University. The experimental protocols were approved by the Animal Ethics Committee of the university (Approval No. YKD202201133), and all procedures were conducted in strict accordance with the regulations of the “Management Measures for Experimental Animals at the Experimental Animal Center of Inner Mongolia Medical University.” For euthanasia, mice were exposed to carbon dioxide (CO_2_) from a compressed gas cylinder at a flow rate of approximately 3 L/min, achieving a displacement rate of 30%–50% of the cage volume per minute using a gradual fill method. The CO_2_ flow was maintained for at least 2 min after respiratory arrest was observed to ensure euthanasia, which was further confirmed by cervical dislocation. Immediately afterward, cardiac blood sampling was performed to collect blood from the heart and aorta, followed by perfusion with phosphate-buffered saline (PBS) for subsequent tissue collection. We randomly selected aortic specimens from three mice in each group for Oil Red O staining (*n* = 3), then randomly selected cardiac specimens from three mice in each group for frozen section analysis (*n* = 3). Blood was left at room temperature for 1 h, then centrifuged at 3,000 rpm for 10 min, the serum supernatant was aspirated with a pipette and finally stored in a refrigerator at −80℃. In order to obtain the organs associated. We extracted the entire aorta, which was subsequently stained in the plaque area of the sinus of the aorta by making a frozen section of the heart for Analysis (*n* = 3). Subsequently, the expression levels and distribution of F4/80, PCSK9 (Proteintech, Wuhan, China, 55206-1-AP), LC3 (Santa Cruz Biotechnology, Dallas, USA, sc-376404), and FOXO3a (Proteintech, Wuhan, China, 66428-1-Ig) proteins within the plaques were analysed. Samples are stored at −80 °C or fixed in 4% PFA. This study was carried out in the Experimental Animal Center of Inner Mongolia Medical University in accordance with the “Regulations for the Administration of Experimental Animals”, this animal study fully complies with the ARRIVE guidelines. The experimental procedure was examined and authorized by the Animal Ethics Committee of Inner Mongolia Medical University (approval number: YKD202201133).

### 2.3 Cell culture and treatment

The Raw264.7 cell line was seeded into 6-well plates at a density of 3.5 × 10^5^ cells per well. Following cell counting, the cells were incubated in DMEM medium supplemented with 10% fetal bovine serum (FBS) and 1% penicillin/streptomycin (P/S) under conditions of 37 °C and 5% CO_2_. Cells were passaged 3–12 times when they reached 80%–90% confluence. Subsequently, the cells were first stimulated with 50 µg/mL ox-LDL for 24 h, and then treated with 200 µg/mL COS for an additional 24 h. The JY2, JY2 and ox-LDL groups were subjected to JY2 post-treatment for 3 h (including 50 µM) in medium.

### 2.4 Small interfering RNA/plasmid transfection of Raw264.7 cells

This study employed Eppendorf centrifuge tubes to mix 100 pmol of PCSK9 siRNA (GenePharma, China, sense: GCACCCAGGUGGAGGUGUAUCUCTT; antisense: GAGAUACACCUCCACCUGGCUGCTT) with 120 μL of OptiMEM, thereby reducing PCSK9 expression. Then, 3 μL of LipoRNAi™ transfection reagent (Beyotime Biotechnology, 384886) was added. The incubation of the mixture was carried out for 25 min. The siRNA/LipoRNAi™ complexes were individually transferred into wells containing Raw264.7 cells, and the processing time was 24 h. PCSK9 expression was increased when the pcDNA3.1-PCSK9 plasmid was used to overexpress PCSK9 (Origene, United States,sense:GAGGATCCCCGGGTACCGGTCGCCACCATGGGC ACCCACTGCTCTGC; antisense: TCCTTGTAGTCCATACCCTGAACCCAGGAGGCCTTTGC). Transfection of the pcDNA3.1 PCSK9 plasmid into the cells was performed following the Lipo8000™ reagent instructions. Twenty-four hours post-transfection, cell lysis was carried out using RIPA buffer. Western blotting was used to detect the transfection efficiency.

### 2.5 RT‒qPCR

Following the protocol of the RNAeasy™ Animal RNA Extraction Kit (Beyotime Biotechnology, Beijing, China), total RNA was extracted from 1 × 106 Raw264.7 cells. The resulting RNA concentration was approximately 0.6 µg/µL, yielding a total mRNA amount of about 24 µg. Take 1 µg of RNA and perform reverse transcription using the PrimeScript™ RT Kit (Takara, RR037B). By SYBR Premix Ex TaqTM kit amplification cDNA (TransGen Biotech, Beijing, China) on an athermocycler. The qPCR reaction program was set as follows: 2-minute pre-denaturation at 95 °C, followed by 40 cycles of 5 s denaturation at 95 °C, 15-second annealing at 60 °C, and 30 s extension at 72 °C. The specific primer sequences are listed in the table below (Supplementary Table S1). Actin was used as a control. Data are expressed as the mean ± standard deviation of at least three determinations of GraphPad Prism. Quantitative analysis was conducted employing the 2^−△△Ct^ calculation method. A value of *p* < 0.05 was considered statistically significant.

### 2.6 Western blot analysis

In order to determine protein concentration, BCA test kit (TransGen Biotech, Beijing, China) was utilized. With the same amount of denatured protein 10% sds-page separation, then transferred to the PVDF membrane. Membranes were blocked for 15 min at room temperature using a rapid blocking solution and then incubated overnight at 4 °C with primary antibodies against PCSK9, FOXO3a, p-FOXO3a, P62, Beclin1, LC3, TLR4, and β-actin. The next day, membranes were washed gently three times with TBST (NaCl, KCl, Tris, Tween). The secondary antibodies, consisting of goat anti-rabbit IgG and goat anti-mouse IgG linked with horseradish peroxidase, were incubated at 37 °C for 1 h. Subsequently, the membrane underwent three more washes with TBST, each lasting 5 min. Specific proteins were visualized via enhanced chemiluminescence kits. ImageJ analysis software with the density measurement method was used to calculate the protein content.

### 2.7 Tandem mRFP-GFP-LC3 assay

A suspension of Raw264.7 cells was evenly distributed into a 24-well plate and maintained in DMEM supplemented with 10% fetal bovine serum (FBS). To assess the steady-state level of cellular autophagy flux following complete ox-LDL stimulation and establishment of the foam cell model, we first treated Raw64.7 macrophages for 24 h. After cell stabilization, re-transfect with ad5-mRFP-GFP-LC3 under ox-LDL conditions for 24 h according to the manufacturer's protocol. The cells were then rinsed three times with PBS. Add serum-containing medium containing si/oePCSK9 to the culture medium and incubate for 36–48 h. Change the medium 6–8 h post-transfection, replacing it with fresh complete DMEM medium for continued culture. Detect mRFP and GFP fluorescence within cells using a push-type confocal microscope (FV1000, Japan). The mean fluorescence intensity (MFl) of acidifiedautophagosomes (RFP+ GFP−) vs. neutral autophagosomes (GFP+ RFP+) per cell werequantified using lmageJ. The data were measured for each treatment condition, and >20 cells were analysed for each populatiom.

### 2.8 Lysotracker staining

The cells were seeded in 24-well plates and exposed to ox-LDL for 24 h. Subsequently, cells were transfected with si/oePCSK9 for 24 h under ox-LDL treatment. They were then stained with LysoTracker Red (Beyotime, C1046) at a concentration of 50 nM for 1 h, followed by three washes with PBS. Red fluorescence within lysosomes was subsequently visualized using laser confocal microscopy.

### 2.9 Masson staining

Aortic sinus frozen sections with PBS washing three times, and then in the paraformaldehyde fixed for 10 min (three consecutive sections from the aortic root were stained and assessed). Following this, Masson's trichrome staining was carried out as per the instructions provided by the manufacturer (Solarbio, G-1346). The sections were stained with mordant solution, placed in a 65 °C incubator for 1 h, stained with celestite blue solution for 3 min, and washed twice. H&E-stained cells were incubated for 3 min, washed with distilled water twice a day, differentiated with acidic differentiation solution for several seconds, and terminated by washing with water. The tissue sections were stained using Ponceau-acid fuchsin solution for a duration of 10 min, after which they underwent two washing cycles with distilled water. Next, the tissue sections were subjected to staining with Mayer's hematoxylin for 3 min, followed by two additional rinses with distilled water, an acetic acid dye solution was used, and a cover slice was added for 2 min. Finally, 95% alcohol and absolute alcohol were added, and the samples were dehydrated and made transparent, with a neutral rubber seal. All histological sections were examined using an inverted microscope (Leica), with images acquired at 4× magnification and subjected to quantitative analysis. Image quantification was performed using ImageJ software for all images. The final data for each animal represented the arithmetic mean of measurements from three analysed sections. Consequently, in the final statistical analysis, each data point (*n*) corresponds to an individual animal (Masson blue-positive area/Total plaque area) × 100%.

### 2.10 Alizarin red S staining

The frozen sections were first air-dried at room temperature and subsequently dried in an oven maintained at 65 °C for a duration of 10 min to ensure complete desiccation (three consecutive sections from the aortic root were stained and assessed). Afterward, the sections underwent three washing steps with PBS. Finally, the samples were treated with alizarin red S solution (pH 4.2) for a duration of 1 h, the samples were rinsed with running water for 5 min, followed by dehydration in 95% and 100% alcohol, each for 10 min. cleared with xylene and sealed with neutral gum to make alizarin red S-stained sections. Calcium salt deposition was observed via an inverted microscope. All histological sections were examined using an inverted microscope (Leica), with images acquired at 10× magnification and subjected to quantitative analysis. Image quantification was performed using ImageJ software for all images. The final data for each animal represented the arithmetic mean of measurements from three analysed sections. Consequently, in the final statistical analysis, each data point (*n*) corresponds to an individual animal.

### 2.11 Immunofluorescence

In a 24-well plate, 70,000 cells per well were inoculated on the cover glass and left to adhere overnight. Raw264.7 cells were then exposed to siPCSK9 (PCSK9 small interfering RNA) and oePCSK9 (PCSK9 overexpression plasmid) for 24 h. After treatment, the medium was renewed and the cells were washed with PBS three times (5 min each). Cells with 4% paraformaldehyde fixed at 37 °C for 30 min, and then use another set of PBS washing. Permeate the cells with 0.2% Triton X-100 in PBS at room temperature for 10 min, and then seal the cells with 2% bovine serum albumin at room temperature for 60 min. Overnight incubation with anti-PCSK9 antibody (diluted 1:200 in PBS) was performed at 4 °C. The next day, add the secondary antibody (diluted at a ratio of 1:200) and DAPI (diluted at a ratio of 1:4), incubate the sample at room temperature for 1 h, followed by three final washes with PBS. Cell visualization was performed using NIS Elements software on a Nikon Eclipse 80i microscope.

For fresh mouse heart tissue samples, tissues were harvested, embedded in OCT compound, frozen, and serially sectioned at low temperatures (three consecutive sections from the aortic root were stained and assessed). The sections were allowed to air-dry for 10–15 min and then underwent permeabilization by being treated with 0.2% Triton-X 100 in PBS for a duration of 10 min. They were then blocked with 10% BSA for 1 h at room temperature. Excess blocking solution was blotted off with filter paper, and sections were incubated with LC3, PCSK9, FOXO3a, the samples were incubated with F4/80 antibodies for a period of 2 h under room temperature conditions. Following three washes with PBS, fluorescent secondary antibodies were added and incubated in the dark at 37 °C for 2 h. Following this, the samples were washed three additional times with PBS. The excess liquid was gently absorbed using filter paper outside the PBS solution, and the sections were finally mounted using fluorescence quenching sealant tablets. NIS software (Elements in Nikon Eclipse 80 I) was used immediately for fluorescence microscope observation. All histological sections were examined using a fluorescence inverted microscope (OLYMPUS). Images were acquired at 20× magnification and subjected to quantitative analysis. Image quantification was performed using ImageJ software for all images. The final data for each animal represented the arithmetic mean of measurements from three analysed sections. Consequently, in the final statistical analysis, each data point (*n*) corresponds to a single independent animal (Fluorescent positive area/Total plaque area) × 100%.

### 2.12 Statistical analysis

The data are expressed as the mean ± standard deviation (SD) from a minimum of three independent measurements. Statistical assessments were performed on GraphPad using one-way ANOVA with appropriate *post hoc* analyses (e.g., Tukey's test) for multi-group comparisons, or two-tailed Student's *t*-tests for two-group comparisons. Where applicable, the values are normalized to the receiving carrier's cell, with the carrier set as the reference value of 1. A *p*-value less than 0.05 was considered statistically significant.

## 3 Results

### 3.1 ox-LDL elevates PCSK9 expression in Raw264.7 cells

**Figure 1:**
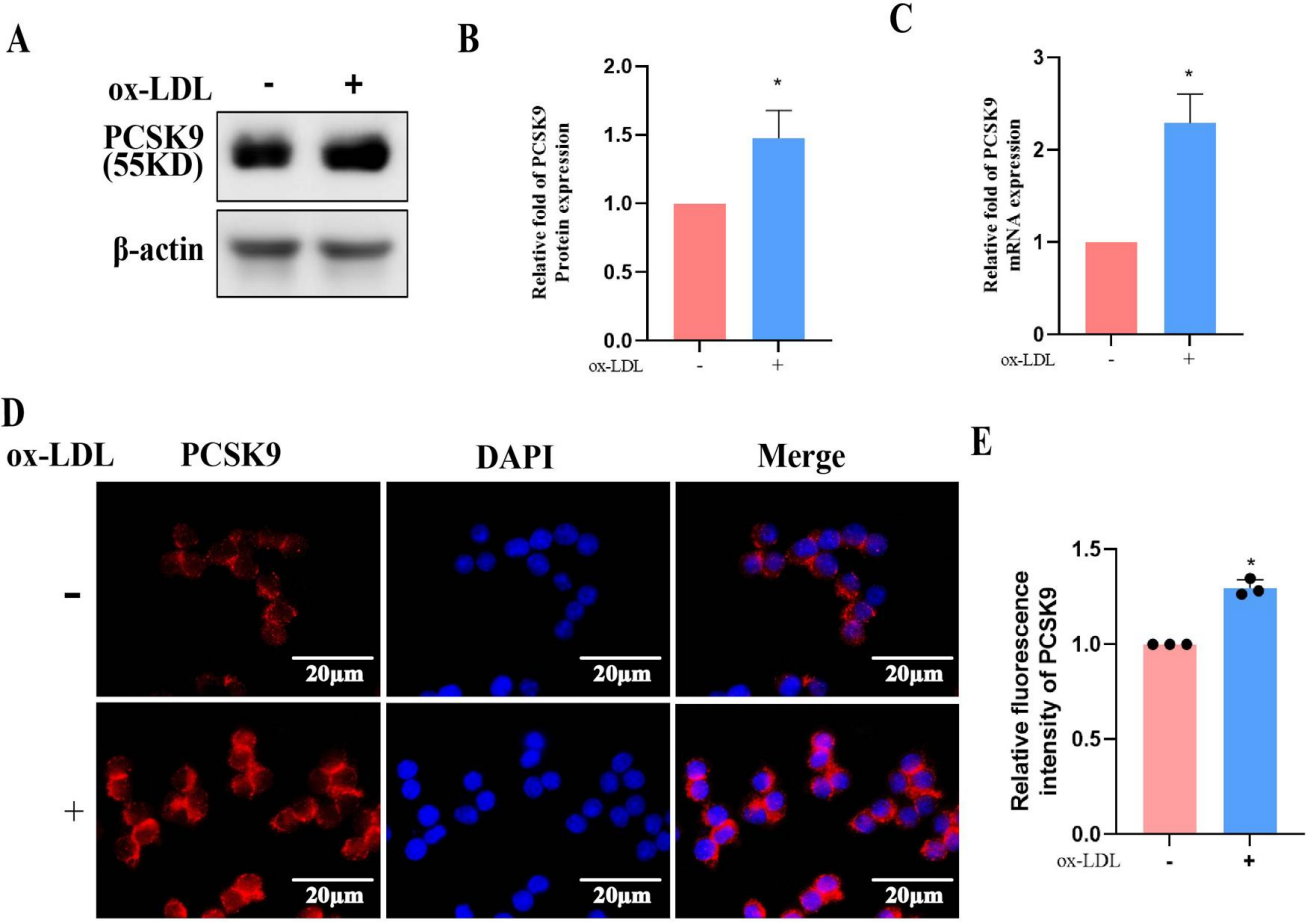
ox-LDL promoted the expression of PCSK9 in Raw264.7 cells. **(A,B)** Western blot detection of PCSK9 expression in Raw264.7 cells following 24 h pre-treatment with ox-LDL (50 µg/mL). **(C)** RT-qPCR detection of PCSK9 gene expression in Raw264.7 cells following 24 h pre-treatment with ox-LDL (50 µg/mL). **(D,E)** Fluorescence intensity of PCSK9 in Raw264.7 cells following 24 h pre-treatment with 50 µg/mL ox-LDL, as determined by immunofluorescence assay (100×). Red: PCSK9 staining; blue: DAPI staining. The bars indicate the mean ± SD. *Values with different superscripts differ significantly (*n* = 3, *p < 0.05 vs. control group, *t* test).

Given that PCSK9 influences the pathogenesis of atherosclerosis, we investigated the potential relationship between PCSK9 and Raw264.7 macrophages. First, we investigated the impact of ox-LDL on Raw264.7 cells. Next, we assessed the expression of PCSK9 in these cells by examining both protein and mRNA levels through western blotting and real-time reverse transcription polymerase chain reaction (RT-PCR), respectively. The findings revealed that treatment with ox-LDL resulted in a substantial increase in PCSK9 protein levels in a time-dependent manner, with the highest expression observed within 24 h (Supplementary Figures S1A,B). Consequently, a 24 h pre-treatment duration was used for subsequent experiments. Incubating Raw264.7 cells with ox-LDL for 24 h led to a significant increase in both PCSK9 protein and mRNA expression levels (Figures 1A–C). PCSK9 expression was further investigated using immunofluorescence, revealing an increase in PCSK9 signal following 24 h ox-LDL treatment (Figures 1D,E). Together, these findings highlight that the PCSK9 gene is expressed in Raw264.7 macrophages and plays a key regulatory role in atherosclerosis progression.

### 3.2 ox-LDL induces heightened autophagic activity in Raw264.7 cells

**Figure 2:**
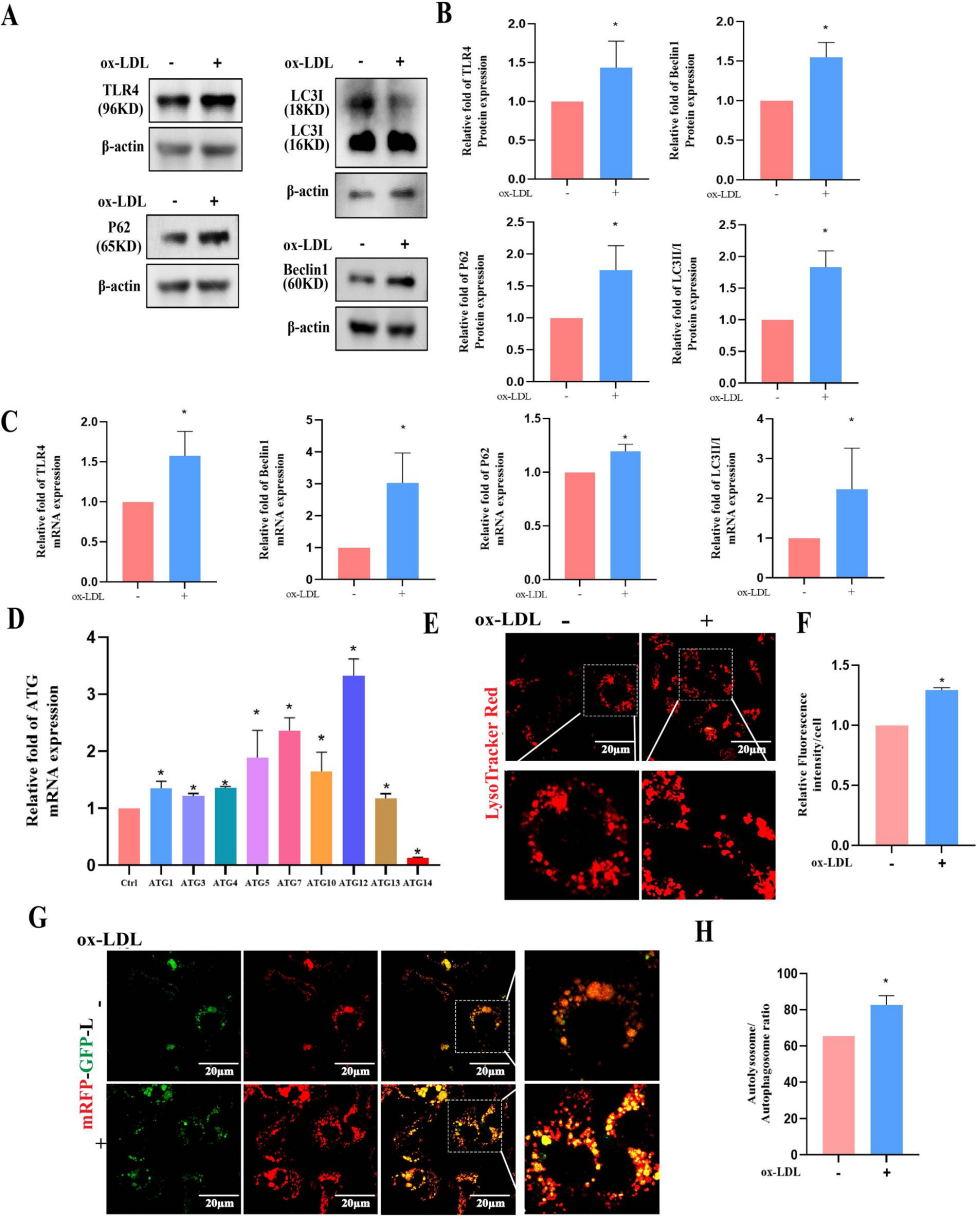
ox-LDL promotes excessive autophagy in Raw264.7 cells. **(A,B)** Western blot detection of TLR4, P62, Beclin1 and LC3 expression in Raw264.7 cells treated with 50 µg/mL ox-LDL for 24 h. **(C,D)** RT-qPCR detection of Expression of autophagy genes in Raw264.7 cells treated with 50 µg/mL ox-LDL for 24 h. **(E,F)** Laser confocal detection of lysosomes in Raw264.7 cells treated with 50 μg/mL ox-LDL for 24 h, red: lysosomes (100×). **(G,H)** Laser confocal detection of autophagosomes in Raw264.7 cells treated with 50 µg/mL ox-LDL for 24 h (100×); yellow dots: autophagosomes or autophagic precursors; red dots: autophagic lysosomes, quantitative analysis based on the red/yellow spot ratio. The bars indicate the mean ± SD. *Values with different superscripts differ significantly (*n* = 3, *p < 0.05 vs. control group, *t* test).

To explore the function of autophagy in Raw264.7 cells more thoroughly, we examined the autophagic activity following ox-LDL treatment. Our findings demonstrated that exposure to ox-LDL markedly increased the expression of Beclin1, Toll-like receptor 4 (TLR4), P62, and LC3 in Raw264.7 cells at both the mRNA and protein levels (Figures 2A–C). Additionally, ox-LDL increased the expression of proteins encoded by autophagy-related genes (ATGs). Specifically, ox-LDL upregulated the mRNA expression of autophagy-related factors ATG1/ULK1 and ATG13 in Raw264.7 cells. Through the ATG1/ULK1 complex, Beclin1 (ATG6) was activated. Among the factors examined, the increase in ATG7 and ATG12 was the most pronounced (Figure 2D); therefore, these key autophagy molecules were selected for further investigation. Furthermore, confocal laser scanning microscopy (CLSM) was used to assess lysosomal content in Raw264.7 cells using a lysosomal red dye. Compared with untreated cells, a notable increase in the number of cells with elevated lysosomal content was observed after 24 h of ox-LDL treatment (Figures 2E,F). In addition, autophagic flux was analyzed using LC3 adenovirus (Ad5–mRFP–GFP–LC3) fluorescence labeling. We observed a significant increase in both autophagosomes (yellow dots, representing autophagosomes or their precursors) and autolysosomes (red dots) in ox-LDL–treated Raw264.7 cells compared with controls. In addition, ox-LDL alone increased autophagic activity during macrophage foam formation (Figures 2G,H). These results indicate that treatment with ox-LDL upregulates the expression of ATG1 and ATG13 in Raw264.7 cells, which in turn activates Beclin1 and its homologous genes (ATG6 and P62), and induces the expression of autophagy assembly genes (ATG5, ATG7, ATG10, and ATG12), facilitating autophagosome processing and transport. Moreover, the expression of ATG14 was inhibited. The activation of ATG3, ATG4, and ATG7 promotes the conversion of LC3-1 to LC3-II, resulting in an increase in Raw264.7 cell autophagy activity. These findings suggest that autophagy in Raw264.7 cells is significantly activated during the atherosclerotic phase.

### 3.3 Modulating PCSK9 expression alters autophagic activity in Raw264.7 cells

**Figure 3:**
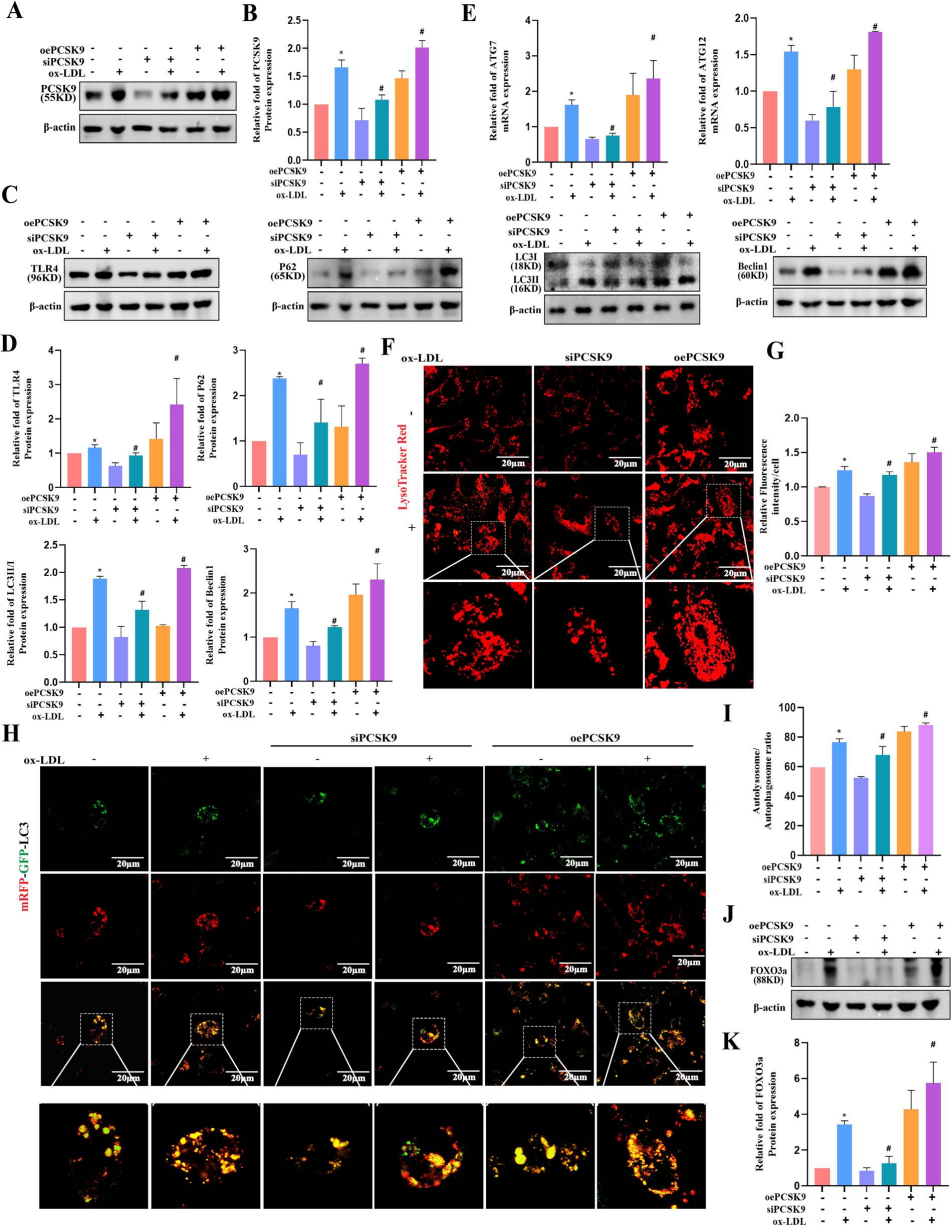
PCSK9 promotes autophagy in foam cells. **(A–D)** Western blot detection of Raw264.7 cells were treated with siPCSK9 or oePCSK9, and PCSK9, TLR4, Beclin1, P62, and LC3 protein levels were detected via Western blot. **(E)** RT-qPCR detection of mRNA expression of ATG7 and ATG12 after siPCSK9 or oePCSK9 treatment of Raw264.7 cells for 24 h. **(F,G)** Laser confocal detection of lysosomal red after siPCSK9 or oePCSK9 treatment of Raw264.7 cells for 24 h, red: lysosomes (100×). **(H,I)** Laser confocal detection of autophagosomes in siPCSK9- or oePCSK9-treated Raw264.7 cells 24 h after treatment (100×), yellow dots: autophagosomes or autophagic precursors; red dots: autophagic lysosomes, quantitative analysis based on the red/yellow spot ratio. **(J,K)** Raw264.7 cells were treated with siPCSK9 or oePCSK9, and FOXO3a protein levels were detected by Western blotting. The bars indicate the mean ± SD. *Values with different superscripts differ significantly (*n* = 3, *p < 0.05 vs. the vehicle group; #*p* < 0.05 vs. the ox-LDL group, *t* test).

Mechanistic studies of cell function revealed that PCSK9 expression in macrophages and atherosclerosis-associated autophagy are closely related. PCSK9 inhibition can significantly improve excessive autophagy in macrophages and autophagy dysfunction during atherosclerosis progression. To investigate the effects of PCSK9 on autophagy in Raw264.7 cells, we established siPCSK9 (silencing) and oePCSK9 (overexpression) models using siRNA and pcDNA3.1-PCSK9 plasmids, respectively. Cells were then exposed to 50 µg/mL ox-LDL for 24 h. The findings indicated that, in comparison to the group treated with ox-LDL alone, the oePCSK9-treated group exhibited markedly elevated protein expression levels of PCSK9, TLR4, Beclin1, P62, and LC3. Conversely, siPCSK9 treatment led to a significant reduction in the expression of these proteins (Figures 3A–D). Furthermore, oePCSK9 significantly elevated the mRNA levels of ATG12 and ATG7, whereas siPCSK9 decreased their expression (Figure 3E). In the oePCSK9-treated group, lysosomal content, indicated by red fluorescence, markedly increased following 24 h of ox-LDL stimulation (Figures 3F,G). In addition, confocal images revealed a decrease in yellow spots in siPCSK9-treated cells and an increase in yellow spots in oePCSK9-treated cells, indicating that oePCSK9 significantly increased the extent of autophagic flux, whereas siPCSK9 significantly reduced the number of autophagosomes (Figures 3H,I). Additionally, FOXO3a protein expression was significantly decreased following siPCSK9 pre-treatment, whereas oePCSK9 increased FOXO3a levels (Figures 3J,K). These findings suggest that PCSK9 may affect atherosclerosis by regulating FOXO3a expression, as its overexpression exacerbates autophagic flux and elevates FOXO3a expression in response to atherosclerotic risk factors.

### 3.4 COS alleviates atherosclerosis by inhibiting PCSK9-mediated autophagy activation

**Figure 4:**
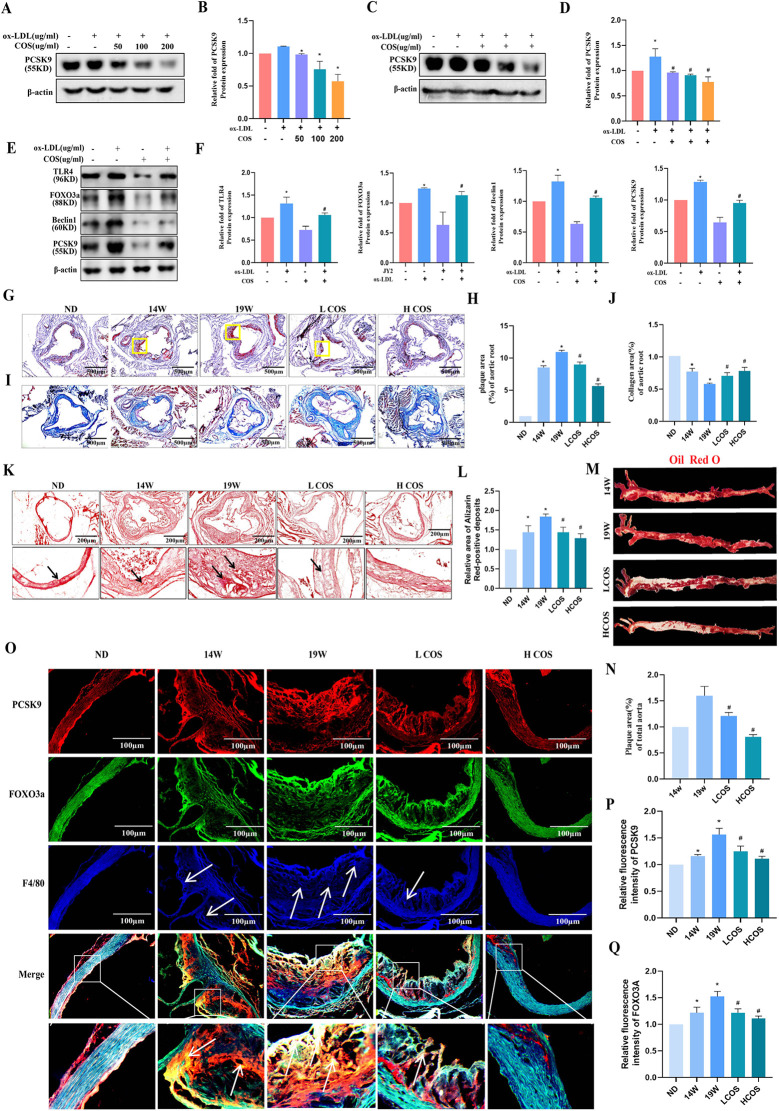
COS downregulates PCSK9 protein expression to inhibit autophagy damage, plaque accumulation and atherosclerosis. **(A,B)** Western blot detection of PCSK9 protein expression in Raw264.7 cells after treatment with COS (200, 100, or 50 μg/mL) for 24 h. **(C,D)** Western blot detection of PCSK9 expression in Raw264.7 cells after 24 h of sequential or simultaneous treatment with 50 μg/mL ox-LDL and COS (200 μg/mL) (Lane 3: COS pretreatment, adding COS first followed by ox-LDL to simulate preventive protection; Lane 4: Combined treatment with COS and ox-LDL; Lane 5: Post-treatment with COS, adding ox-LDL first followed by COS to simulate therapeutic intervention). **(E,F)** Western blot detection of PCSK9 expression in Raw264.7 cells after treatment with 50 µg/mL ox-LDL for 24 h followed by treatment with COS (0, 200 µg/mL) for 24 h, FOXO3a, Beclin1, and TLR4 expression. **(G,H)** Plaque area after oil red O staining in frozen sections of mouse aortic orifices [(Oil red O-positive area/Total plaque area) × 100%, 4×, *n* = 3]. **(I,J)** Area of collagen fibers in Masson-stained frozen sections of mouse aortic orifices [(Area of blue collagen/Reference total area) × 100%, 4×, *n* = 3]. **(K,L)** Amount of calcium deposits in alizarin red-stained frozen sections of mouse aortic orifices [(Alizarin red positive area/Total vascular area) × 100%, 10×, *n* = 3]. **(M,N)** Plaque area after oil red O staining in longitudinal sections of the mouse aorta [(Plaque area/Total vascular lumen area) × 100%, *n* = 3]. **(O–Q)** Immunofluorescence detection of PCSK9 (red), FOXO3a (green) and F4/80 (blue) expression in frozen sections of mouse aortic orifices (20×, *n* = 3). The bars indicate the mean ± SD. *Values with different superscripts differ significantly (*n* = 3, *p < 0.05 vs. the vehicle group or ND group; #*p* < 0.05 vs. the ox-LDL group or 19W group, *t* test).

Our previous study revealed that COS effectively alleviates atherosclerosis in ApoE−/− transgenic mice (35), however, its effects on PCSK9-mediated autophagy and atherosclerosis need to be further explored. To further investigate the role of PCSK9 on autophagy, we used COS as an inhibitor of PCSK9 to explore the effect of PCSK9 on autophagy, and analyzed PCSK9 expression in ox-LDL-induced Raw264.7 macrophages. Our results indicated that COS reduced the protein expression of PCSK9 in a dose-dependent manner, with the lowest levels observed at 200 µg/mL (Figures 4A,B). In addition, PCSK9 expression was significantly reduced in COS post-treatment cells compared to both the ox-LDL-treated group. These results suggest that COS can produce a favorable therapeutic effect on atherosclerosis by inhibiting PCSK9 expression (Figures 4C,D). Therefore, we subsequently opted for the COS post-treatment approach to investigate the molecular mechanisms of autophagy in the atherosclerotic process. To elucidate the potential direct mechanism by which COS inhibits PCSK9, molecular docking analysis was performed. These results indicate a strong binding affinity between COS and PCSK9 (Supplementary Figures S2A,B). Furthermore, COS post-treatment significantly improved lipid levels in atherosclerosis model mice (Supplementary Figures S2C,D). Additionally, our results demonstrated that COS markedly reduced the protein expression of FOXO3a, Beclin1, and TLR4 in ox-LDL-treated cells (Figures 4E,F), indicating that COS can inhibit autophagy by targeting PCSK9. Specifically, the optimal conformation of COS bound to PCSK9 yielded a -CDOCKER_ENERGY score of −2.19494. The binding pocket was defined at coordinates (X: 16.74, Y: 34.11, Z: −24.68), with specific hydrogen bond interactions identified at amino acid residues ASN A:533, SER A:535, and GLN A:554.

Given the critical role of COS in atherosclerotic plaque formation through autophagy, we assessed its impact on plaque formation in ApoE−/− mice. To evaluate atherosclerotic lesions in the aortic root, we utilized Oil Red O staining. In frozen sections from ApoE−/− mice, 19W lipid deposition was typically observed in the aortic sinus; however, it was significantly reduced in the high-dose COS (HCOS) treatment group (Figures 4G,H). In addition, Masson's three colors and alizarin red staining revealed that in 19W ApoE−/− mice, calcium salt deposits were significantly increased in the aortic plaques, whereas thin fibrous caps and collagen fibers were reduced. In contrast, compared with the 19W group, LCOS or HCOS treatment increased collagen fiber content and reduced calcium salt deposition (Figures 4I–L). Furthermore, aortic Oil red O staining revealed that both LCOS and HCOS significantly decreased the plaque area in ApoE−/− mice compared to the 19W group (Figures 4M,N). Therefore, low-dose COS (LCOS) or HCOS treatment can significantly reduce plaque size and inhibit lipid accumulation within plaques. Importantly, fluorescence intensities of PCSK9 and FOXO3a in aortic plaques also changed significantly. Compared with those in 19W group, the fluorescence intensities of PCSK9 and FOXO3a in the HCOS group were significantly lower (Figures 4O–Q). These results suggest that COS-mediated downregulation of PCSK9 not only inhibits autophagy-related targets and the progression of atherosclerosis in ApoE−/− mice, but also inhibits FOXO3a expression in macrophages. Thus, COS can inhibit autophagic injury and atherosclerosis progression in ApoE−/− mice by downregulating PCSK9/FOXO3a autophagy pathway.

### 3.5 The PCSK9-mediated FOXO3a autophagy signaling pathway can be suppressed by COS to slow the progression of atherosclerosis

**Figure 5:**
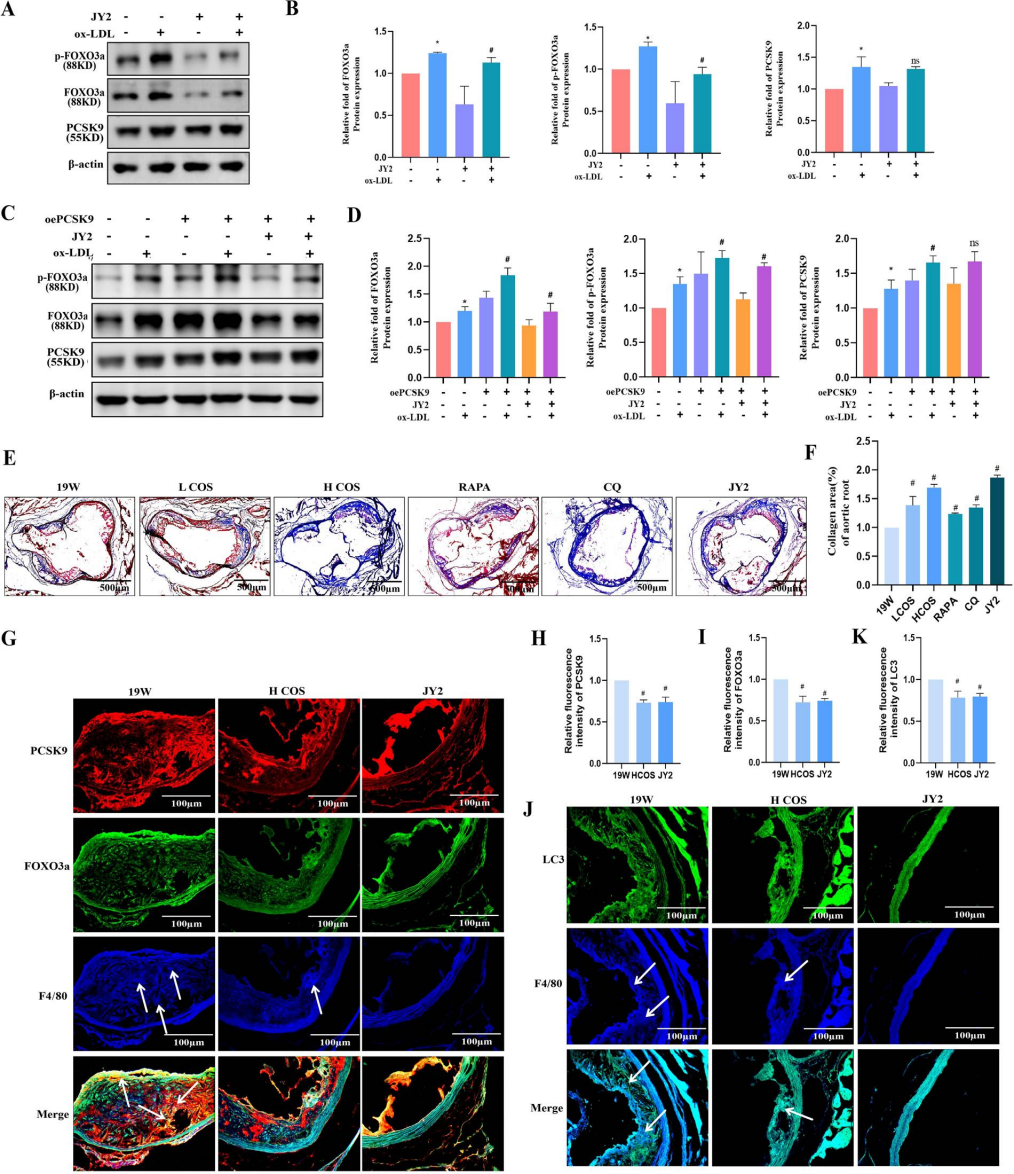
COS inhibits the PCSK9-mediated FOXO3a autophagy pathway and reduces the development of atherosclerosis. **(A,B)** Western blot detection of FOXO3a expression, p-FOXO3a and PCSK9 in Raw264.7 cells after post-treatment with JY2 (10 μM) for 24 h. **(C,D)** After Raw264.7 cells were treated with siPCSK9 or oePCSK9 for 24 h, Western blot detection of FOXO3a, p-FOXO3a and PCSK9 expression was detected after JY2 (10 μM) post-treatment for 24 h. **(E,F)** Collagen fiber area of Masson-stained frozen sections of mouse aortic orifices [(Area of blue collagen/Reference total area) × 100%, 4×, *n* = 3]. **(G–I)** Fluorescence intensity of FOXO3a (green), PCSK9 (red) and F4/80 (blue) after immunofluorescence staining of frozen sections of mouse aortic orifices (20×, *n* = 3). **(J,K)** Fluorescence intensity of LC3 (green) and F4/80 (blue) after immunofluorescence staining of frozen sections of mouse aortic orifices (*n* = 3). The bars indicate the mean ± SD. *Values with different superscripts differ significantly (*n* = 3, *p < 0.05 vs. the vehicle group; #*p* < 0.05 vs. the ox-LDL group or 19W group, ns*p* > 0.05 vs. ox-LDL group, test).

These studies indicate that COS may reduce atherosclerotic plaque development by inhibiting autophagic flux and thereby diminishing excessive autophagy in Raw264.7 cells. To determine if the FOXO3a/autophagy signaling pathway is modulated by PCSK9 in Raw264.7 cells, we examined the effects of the FOXO3a-specific inhibitor JY2 on FOXO3a expression using Western blotting. Our results demonstrated that JY2 markedly reduced the protein expression of phosphorylated FOXO3a (p-FOXO3a) and total FOXO3a, with no significant change observed in PCSK9 levels (Figures 5A,B). Western blot analysis revealed that JY2 post-treatment significantly inhibited oePCSK9-induced increase in p-FOXO3a and FOXO3a protein levels without affecting PCSK9 expression (Figures 5C,D). Additionally, JY2 post-treatment alleviated the upregulation of the FOXO3a/autophagy signaling pathway induced by oePCSK9. These findings suggest that PCSK9 exacerbates atherosclerosis by regulating autophagy dysfunction mediated through FOXO3a. *In vivo* experiments employed rapamycin (RAPA) as a positive control to promote late autophagy, chloroquine (CQ) as a negative control to inhibit late autophagy damage, and JY2 post-treatment to further validate COS's regulatory effects on PCSK9 and FOXO3a. Results showed that ApoE−/− mice treated with HCOS exhibited significantly reduced calcification and plaque accumulation compared to the RAPA group (Supplementary Figures S3A–D), and the fluorescence intensities of PCSK9 and FOXO3a in aortic plaques were markedly reduced (Supplementary Figures S3E–G). These findings further indicate that COS can inhibit the progression of atherosclerosis and is associated with the PCSK9/FOXO3a autophagy pathway. We subsequently used Masson trichrome staining to investigate whether FOXO3a induces atherosclerotic progression in ApoE−/− mice. Compared with the 19W group, the aortic sinus fibrous cap was thinner and collagen fibers were relatively increased in both the HCOS and JY2 groups (Figures 5E,F). To further characterize the interaction between the PCSK9, FOXO3a autophagy pathway, and atherosclerosis, we performed tissue immunofluorescence analysis. Our results indicate that in the JY2-treated group, the fluorescence intensity of PCSK9 and FOXO3a in the aortic sinus region of ApoE−/− mice was significantly reduced compared to the 19W group. Similarly, LC3 expression exhibited a consistent pattern (Figures 5G–K). The aforementioned findings indicate that both COS and JY2 are capable of reducing the levels of FOXO3a and PCSK9, which in turn can suppress the levels of the downstream autophagy key target LC3, enhance the stability of plaques, reduce autophagy disorders, and slow down atherosclerosis. To summarize, the collective findings underscore the involvement of PCSK9 in facilitating atherosclerosis through FOXO3a-mediated autophagy dysregulation, and these effects can be reversed by COS.

## 4 Discussion

Atherosclerosis is characterized by foam cell accumulation within arterial walls, driven primarily by intracellular cholesterol and lipid droplet overload (39–44). Clinical studies demonstrate that PCSK9 inhibition effectively lowers LDL-C and reduces cardiovascular risk. PCSK9 also activates macrophages and hepatocytes to release proinflammatory cytokines, and modulates TLR4 expression, thereby influencing the NF-*κ*B signaling pathway, apoptosis, and autophagy (45–47). Our previous research indicates that chitosan oligosaccharide (COS) attenuates atherosclerotic progression by suppressing PCSK9 expression (48, 49). Here, we demonstrate that COS inhibits excessive autophagy through the FOXO3a signaling pathway, thereby slowing atherosclerotic progression.

In the present study, PCSK9 overexpression in Raw264.7 cells upregulated LC3, Beclin1, p62, FOXO3a, p-FOXO3a, and TLR4 protein levels. Both PCSK9 knockdown and COS post-treatment significantly suppressed Beclin1 expression. Beclin1 is a critical initiator of autophagosome formation and a key regulator of autophagy development (50). Confocal microscopy further confirmed altered autophagic flux in PCSK9-modulated cells. These findings suggest that reducing PCSK9 expression or COS treatment may prevent autophagosome accumulation, potentially halting advanced atherosclerosis progression. However, this mechanism remains speculative, as direct functional evidence linking autophagy markers to inhibitor activity is currently lacking. Moreover, siRNA-mediated knockdown achieved only partial gene silencing, resulting in relatively modest downstream phenotypic changes. Therefore, the identified association should be regarded as preliminary evidence requiring validation with more efficient genetic tools in future studies.

We further demonstrate that PCSK9 promotes autophagy and atherosclerosis progression in Raw264.7 macrophages by enhancing the FOXO3a/autophagy signaling pathway. Using rapamycin (RAPA) and chloroquine (CQ) as positive and negative controls for autophagic flux, we systematically evaluated plaque formation, lipid deposition, and calcification. The FOXO transcription factor inhibitor JY2—which exhibits moderate inhibitory activity against FOXO3a and FOXO4 (51)—downregulated FOXO3a and inhibited autophagy without altering PCSK9 expression, suggesting that FOXO3a functions downstream of PCSK9. FOXO3a activation of autophagy plays a critical role in coordinating cellular redox homeostasis (52). In ApoE^−/−^ mice, JY2 treatment significantly reduced atherosclerotic plaque area and lipid deposition while increasing collagen content. These findings confirm the critical role of the PCSK9/FOXO3a autophagy pathway in atherosclerosis progression and support its potential as a therapeutic target. Recent studies also suggest that macrophage lipid metabolism can actively remodel the stromal compartment and drive fibroblast expansion (53), and this mechanism may underlie the collagen and fibrous cap changes we observed. COS similarly attenuated atherosclerosis by inhibiting this pathway, providing a novel perspective on macrophage autophagy regulation in plaques and establishing a foundation for developing targeted therapeutic strategies.

This study has several directions that warrant further investigation. First, although JY2 effectively modulates FOXO3a activity, subsequent work needs to incorporate direct binding assays (such as ChIP, luciferase reporter, or co-immunoprecipitation) to elucidate the precise transcriptional mechanisms by which FOXO3a regulates autophagy genes. Second, although the Raw264.7 cell line provides a robust platform for genetic manipulation, these findings still need to be extended to primary macrophages (such as bone-marrow-derived macrophages, BMDMs) to further enhance physiological relevance. In addition, we recognize that expanding the histological sample size (beyond *n* = 3) will increase the statistical power for lesion quantification, which we plan to carry out in follow-up studies. Although molecular docking analysis has identified the specific interactions between COS and PCSK9, future experiments incorporating comparator compounds and DMSO vehicle controls will further validate these computational predictions. Finally, our focus on PCSK9-FOXO3a and autophagy opens promising research directions for exploring its interplay with macrophage efferocytosis and epigenetic regulators. Integrating these dimensions into future research frameworks will facilitate a more comprehensive understanding of the dynamic evolution of atherosclerotic plaques.

## Data Availability

The original contributions presented in the study are included in the article/Supplementary Material, further inquiries can be directed to the corresponding authors.

## Supplementary material

Supplementary Figure S1 PCSK9 expression time course.

Supplementary Figure S2 COS-PCSK9 docking and lipid data.

Supplementary Figure S3 Plaque calcification and PCSK9/FOXO3a fluorescence.

Supplementary Table S1 Primer sequences.
