# Supplementary figures and images for: PCSK9 promotes atherosclerosis progression through the FOXO3a autophagy signaling pathway
Source: Front Cardiovasc Med. 2026 Jun 15;13:1836294. doi: 10.3389/fcvm.2026.1836294 (PMC13310736; doi:10.3389/fcvm.2026.1836294)

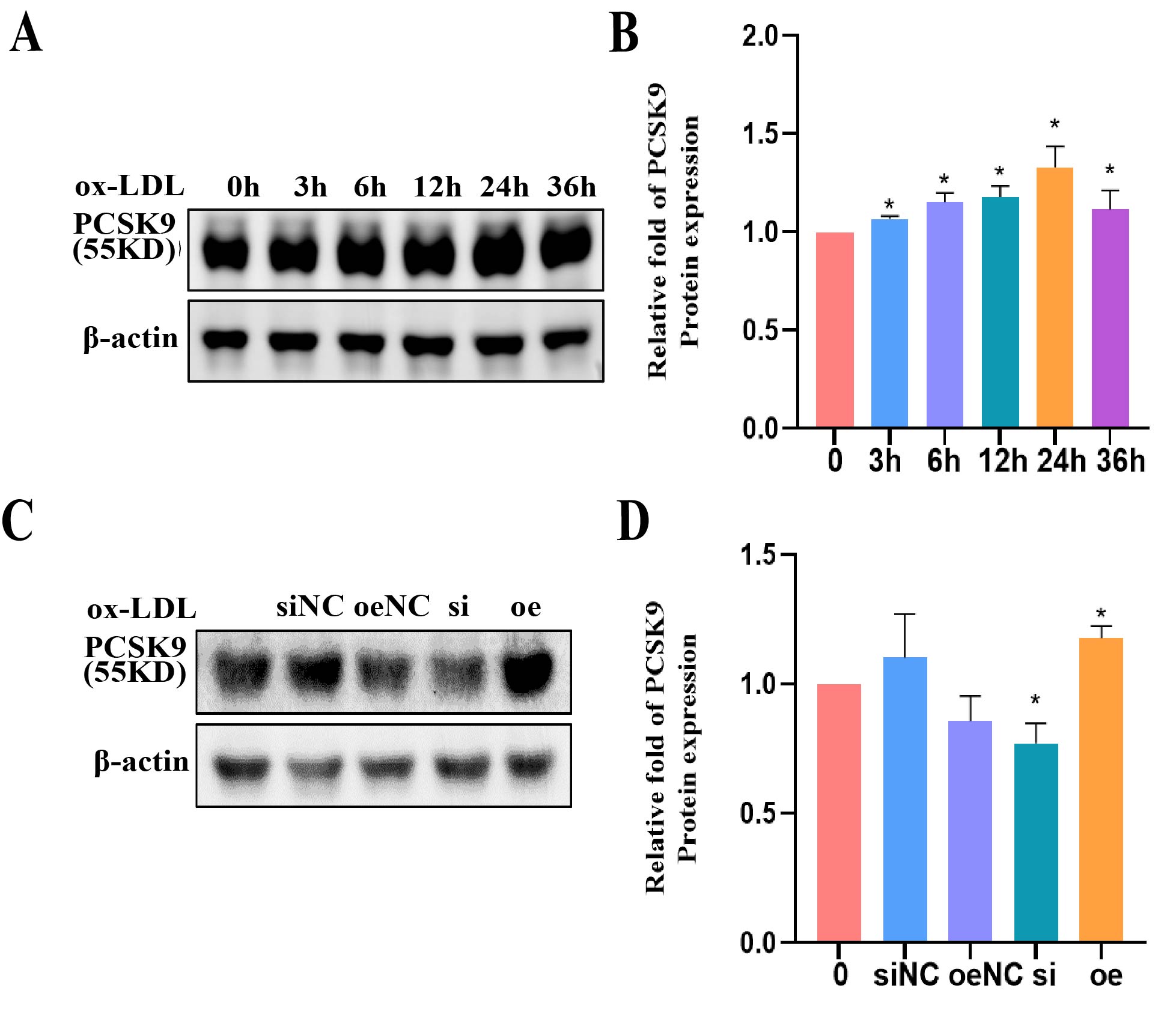

Supplement: Supplementary Figure S1 — PCSK9 expression time course. [file Image1.jpg]

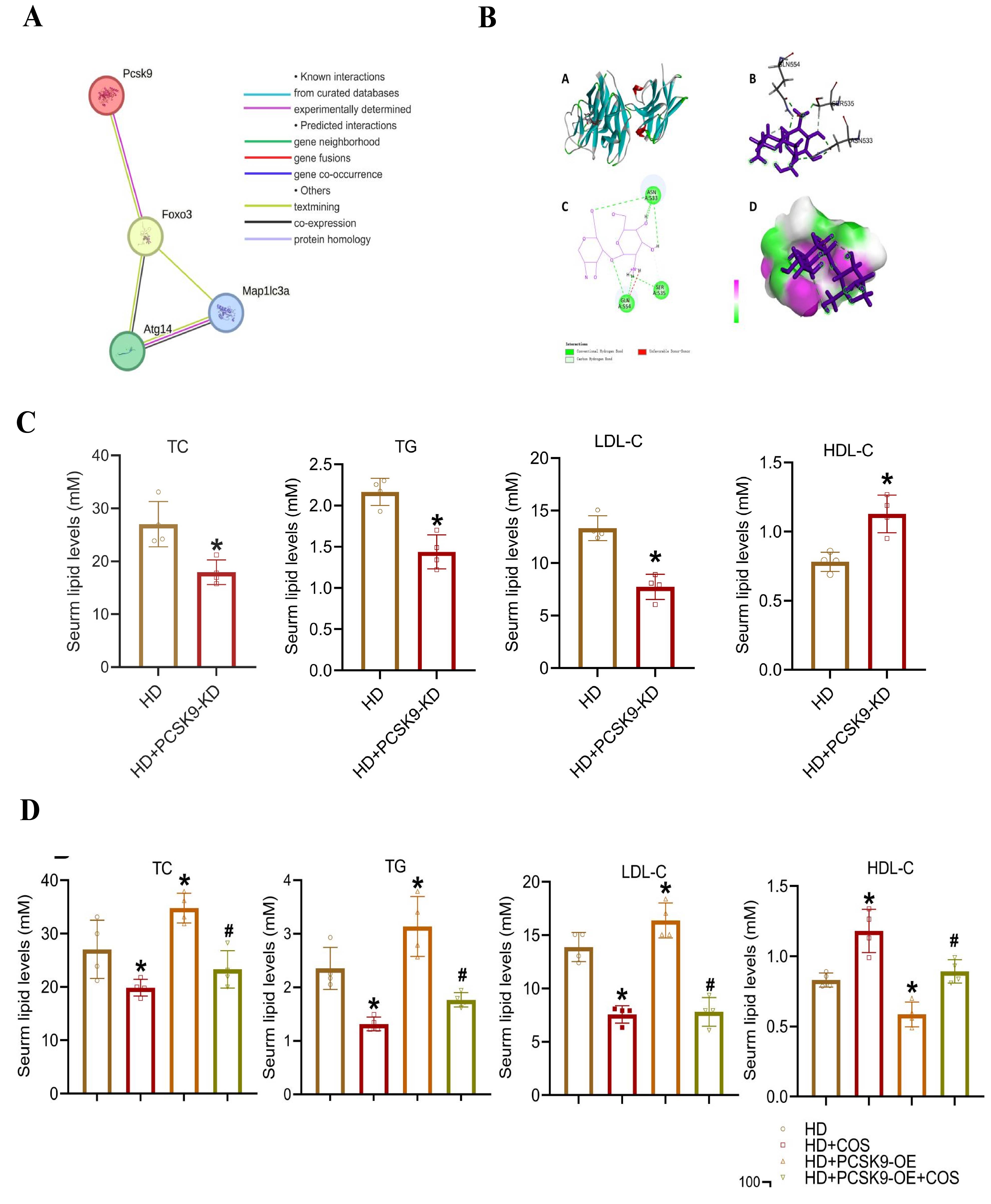

Supplement: Supplementary Figure S2 — COS-PCSK9 docking and lipid data. [file Image2.jpg]

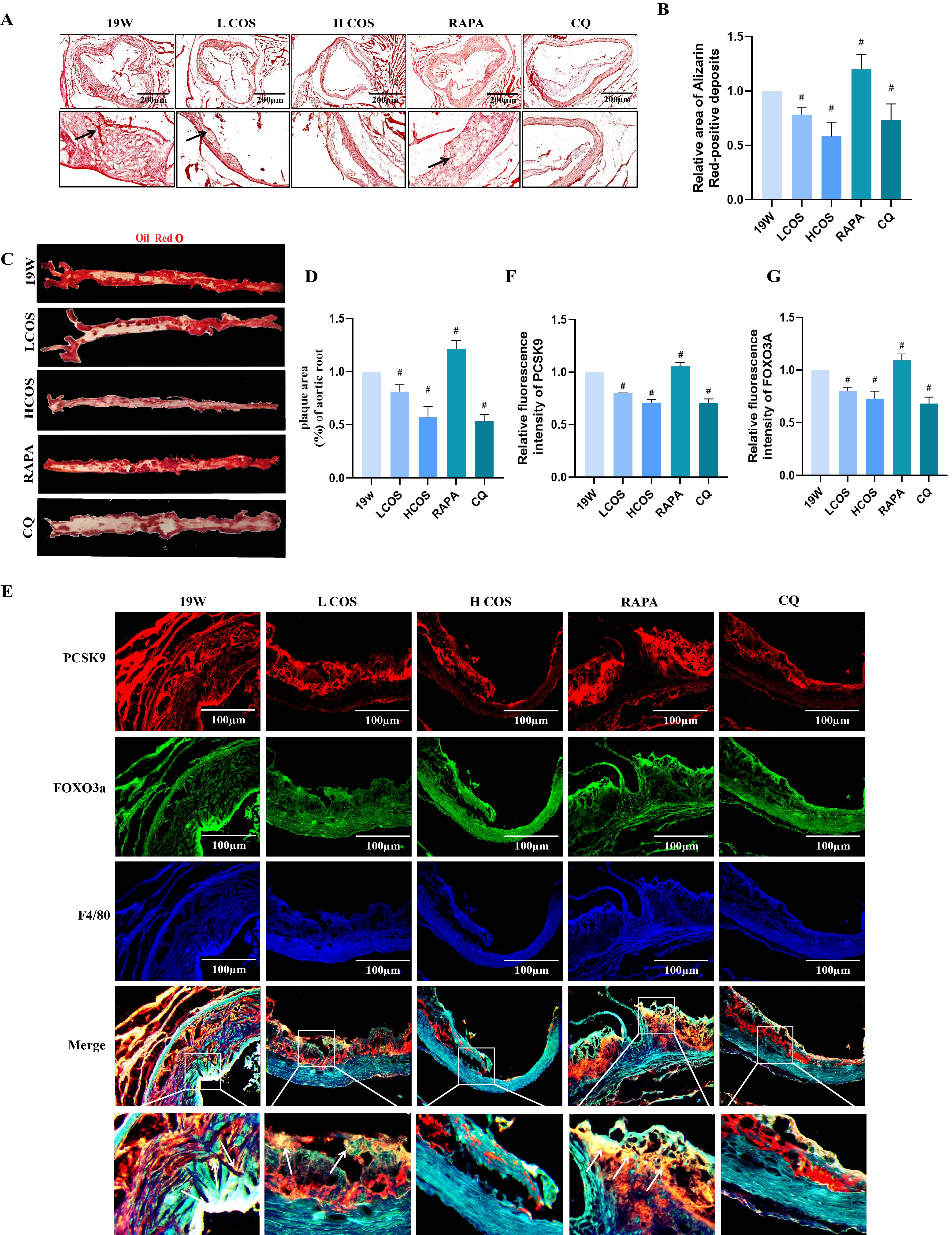

Supplement: Supplementary Figure S3 — Plaque calcification and PCSK9/FOXO3a fluorescence. [file Image3.jpg]
