# Supplementary material for: PCSK9 promotes atherosclerosis progression through the FOXO3a autophagy signaling pathway
Source: Front Cardiovasc Med. 2026 Jun 15;13:1836294. doi: 10.3389/fcvm.2026.1836294 (PMC13310736; doi:10.3389/fcvm.2026.1836294)
Supplement: Supplementary Table S1 — Primer sequences. [file Datasheet1.docx]

Supplementary Tabel S1

| Gene name | Primer sequence (5′-3′) |
| --- | --- |
| PCSK9-F | ACCATACAGGACTTACCGC |
| PCSK9-R | CTGGGGGCTAAGGGAGTAT |
| LC3-F | GGAGTCCTGTGTCTACGG |
| LC3-R | AAAAGCTGGGGTGTTCCT |
| FOXO3a-F | CCTACTTCAAGGATAAGGGCGAC |
| FOXO3a-R | GCCTTCATTCTGAACGCGCATG |
| Beclin1-F | CTGTGCATTCCTCACACAGC |
| Beclin1-R | CCCAGCCAGGATGATGTCTA |
| P62-F | ATGTGGAACATGGAGGGAAGA |
| P62-R | GGAGTTCACCTGTAGATGGGT |
| TLR4-F | ATGGCATGGCTTACACCACC |
| TLR4-R | GAGGCCAATTTTGTCTCCACA |
| ATG1-F | CAGCAAAGACTCCTGTGACAC |
| ATG1-R | CCACTACACAGCAGGCTATCAG |
| ATG3-F | TAAGGCTGACGCTGGAGGTGAA |
| ATG3-R | GTGCTCAACTGTTAAAGGCTGCC |
| ATG4-F | CAGTCTCCACAGCGGATGATGA |
| ATG4-R | GTGTGATGGGTGCTTCTGAACC |
| ATG5-F | AGTCTGTCCTTCCGCA |
| ATG5-R | GTCACGCCTCGTTGTC |
| ATG10-F | GAGACCTTGACACCACATGCCA |
| ATG10-R | CAGGTCTCGTCACTTCAGAATC |
| ATG12-F | CTTCTGGGCCTGCTGTTCACAGT |
| ATG12-R | TTCTTGGGGTCAGCACAGACCTC |
| ATG13-F | AGAGACTGGTGATGCACATGCC |
| ATG13-R | CCGTCCTTCACTGCTGTTAGAC |
| ATG14-F | ATCTTCTTGTGCAGTGCCAGCCTC |
| ATG14-R | TTTGCCACTGCAAATGGCAGCC |
| β-actin-F | AAGACCTCTATGCCAACAC |
| β-actin-R | CTGCTTGCTGATCCACA |
